# Prognostic value of noninvasive programmed stimulation in primary prevention implantable cardioverter‐defibrillator recipients

**DOI:** 10.1002/joa3.13017

**Published:** 2024-03-15

**Authors:** Piotr Futyma, Pasquale Santangeli, Łukasz Zarębski, Aleksandra Wrzos, Jarosław Sander, Marian Futyma, Francis E. Marchlinski, Piotr Kułakowski

**Affiliations:** ^1^ St. Joseph's Heart Rhythm Center Rzeszów Poland; ^2^ Medical College University of Rzeszów Rzeszów Poland; ^3^ Section of Cardiac Pacing and Electrophysiology, Heart and Vascular Institute Cleveland Clinic Foundation Cleveland Ohio USA; ^4^ Clinical Electrophysiology Hospital of the University of Pennsylvania Philadelphia Pennsylvania USA; ^5^ Department of Cardiology, Centre of Postgraduate Medical Education Grochowski Hospital Warsaw Poland

**Keywords:** implantable cardioverter‐defibrillator, noninvasive programmed ventricular stimulation, primary prevention, prognostic value, ventricular arrhythmias

## Abstract

**Background:**

Implantable cardioverter‐defibrillator (ICD) offers an opportunity to study inducibility of ventricular tachycardia (VT) or ventricular fibrillation (VF) by performing noninvasive programmed ventricular stimulation (NIPS). Whether NIPS can predict future arrhythmic events or mortality in patients with primary prevention ICD, has not yet been examined.

**Methods:**

From the NIPS‐ICD study (ClinicalTrials ID: NCT02373306) 41 consecutive patients (34 males, age 64 ± 11 years, 76% ischemic cardiomyopathy [ICM]) had ICD for primary prevention indication. Patients underwent NIPS using a standardized protocol of up to three premature extrastimuli at 600, 500 and 400 ms drive cycle lengths. NIPS was classified as positive if sustained VT or VF was induced. The study endpoint was occurrence of sustained VT/VF during the follow‐up.

**Results:**

At baseline NIPS, VT/VF was induced in 8 (20%) ICM patients. During the 5‐year follow‐up, the VT/VF occurred in 7 (17%) patients, all with ICM. The difference between NIPS‐inducible versus NIPS‐noninducible patients regarding VT/VF occurrence did not meet statistical significance (38% vs. 12%, log rank test *p* = .11). After a 5‐year follow‐up, the mortality rate was significantly higher in patients who had VT/VF induced at NIPS versus no VT/VF at NIPS (38% vs. 12%, *p* = .043). The occurrence of a composite endpoint consisting of VT/VF recurrence or death in patients with ICM was also most frequent in the NIPS‐inducible group (75% vs. 35%, *p* = .037).

**Conclusions:**

Inducibility of VT/VF during NIPS in ICM patients with primary prevention ICD is associated with higher mortality and higher incidence of composite endpoint consisting of death or VT/VF during a long‐term observation.

## Graphical abstract

Whether NIPS can predict future arrhythmic events or mortality in patients with primary prevention ICD, has not yet been examined. The NIPS‐ICD study indicates that inducibility of VT/VF during NIPS in patients with primary prevention ICD is associated with higher mortality and higher incidence of composite endpoint consisting of death or VT/VF during a long‐term observation.

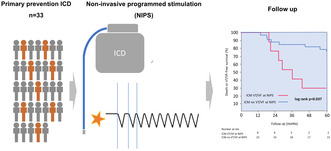

## 1 INTRODUCTION

Therapy delivered from implantable cardioverter‐defibrillator (ICD) can protect from sudden cardiac death caused by ventricular tachycardia (VT) or ventricular fibrillation (VF). Occurrence of such life‐saving appropriate ICD interventions is lower in patients who had primary prevention indication for ICD.^1^ When these therapies occur, ICD interventions can be painful and have negative prognostic value.^2^ Additionally, ICD benefit does not extend to causes other than VT/VF such as heart failure (HF) acceleration. Modern strategies of management of patients with ICD include proper ICD programming, catheter ablation, treatment with antiarrhythmic or HF drugs.^3^ However, prophylactic catheter ablation is rarely performed whereas the effectiveness of antiarrhythmic drugs is restricted and may be associated with adverse events. Thus, methods which could predict occurrence of VT/VF or death in patients already equipped with ICD so that they can potentially benefit from prophylactic treatment.

One approach to predict future events is ventricular noninvasive programmed stimulation (NIPS). A few studies suggested that appropriate interventions occur more frequently in patients who had VT/VF induced by NIPS, however data from prospective trials on significance of NIPS is limited.^4^, ^5^, ^6^, ^7^ Previously we reported the 1‐year outcomes in the whole cohort of patients included in the NIPS‐ICD study.^7^ Herein, we present data on the 5‐year follow‐up in a cohort with ICD implanted for primary prevention.

## 2 METHODS

The NIPS‐ICD study protocol was approved by the Ethics Committee of the Regional Medical Association. The study was registered (ClinicalTrials.gov ID: NCT02373306) and detailed study protocol was published.^8^ The NIPS‐ICD is a single‐center study which included patients with their ICD implanted both for primary and secondary prevention indication. The aim of the current analysis is to describe the 5‐year observation in the primary prevention NIPS‐ICD subgroup.

The current study included patients with primary prevention who attended our outpatient clinic and who did not meet the following exclusion criteria: lack of consent for NIPS, decompensated heart failure NYHA class IV, unstable angina, persistent/long standing AF without effective anticoagulation, thrombus in LV, prior appropriate ICD interventions or pacing/sensing problems. None of the patients in the current study group had neither sustained VT nor VF prior to ICD implant or prior to NIPS. None of the patients underwent catheter ablation prior to ICD implant or prior to NIPS. Dominant cardiac condition was ischemic cardiomyopathy (ICM) in 31 patients and nonischemic cardiomyopathy (NICM) in the remaining 10 patients. After inclusion, patients were scheduled for NIPS. Patients were admitted in the fasting state. After history taking and physical examination, patient was informed about the manner and purpose of the NIPS, and written consent was obtained. The ECG was recorded and access to the venous system was obtained. The patient was transferred to the EP room. Throughout the duration of the NIPS, surface 12‐lead ECG using the electrophysiological system (EP Tracer, Cardiotek, Maastricht, the Netherlands) was recorded. ICD was interrogated and all available events and interventions stored in the ICD memory were analyzed. Also, the low and high voltage impedance, the signal amplitude from the defibrillation lead, and the pacing threshold were examined.

Next, each patient underwent a 12‐step stimulation protocol using ICD,^9^ which consisted of introduction of single, double and triple extrastimuli, delivered with decreasing coupling intervals up to ventricular refractory period or 200 ms, from the ICD right ventricular lead during sinus rhythm (or atrial fibrillation if present) and after eight‐beat drive trains at paced cycle lengths of 600, 500, and 400 ms. The endpoint of NIPS was induction of sustained ventricular arrhythmia (VT lasting more than 30 s or hemodynamically unstable VT/VF) or the end of the protocol. The stage of NIPS at which arrhythmia was induced and the type of arrhythmia were analyzed. In case of VT induction mean VT cycle length (calculated from the first 10 QRS complexes of stable VT), effectiveness of anti‐tachycardia pacing (ATP) in terminating arrhythmia, and, in case of ineffectiveness of ATP—efficacy of internal cardioversion/defibrillation, were examined. In case of serious and/or hemodynamically unstable rhythm resistant to low‐energy ICD therapy, internal or external defibrillation was performed. If VT/VF was induced by NIPS, a patient, after sinus rhythm restoration, was transferred to the intensive care unit, where continuous ECG and blood pressure were monitored. No changes in medications were done regarding the result of NIPS.

During first 12 months after NIPS, the follow‐up was conducted every 3 months at the outpatient clinic located in our center. After 1 year, follow‐up visits were scheduled individually. The end‐point of the follow‐up was death or appropriate intervention of ICD due to VT/VF. Records from the device memory classified as VT/VF were collected in printed or electronic versions for further investigation by the study team. Each collected episode was examined independently by two study members, of which one was blinded to the result of NIPS.

Follow‐up visits were combined with taking patients medical history and physical examination: the patient's clinical status was determined on the basis of physical examination, NYHA class, changes in medication, hospitalizations, cardiac events such as syncope, ICD interventions, palpitations and other symptoms, which occurred after the previous visit. In case of reaching the follow‐up end‐point, the time from NIPS test to the first ICD episode detection/therapy or death was used for statistical analysis.

### 2.1 Statistical methods

The results are presented as mean ± SD or numbers and percentages. Continuous variables were compared using two‐tailed Student's *t*‐test or Mann–Whitney test where appropriate. Qualitative variables were compared using Chi square test with Yates correction or Fisher exact test where appropriate. The principal analysis examined the relationship between positive NIPS result (VT/VF induction) and the follow‐up outcome. The occurrence of endpoints during the follow‐up in the NIPS‐positive and NIPS‐negative groups was compared using Kaplan–Meier survival curves and tested with a log‐rank test. The sensitivity, specificity, positive, and negative predictive values of NIPS in identification of patients who experienced VT/VF or death during the follow‐up were calculated using standard formulas.

## 3 RESULTS

### 3.1 NIPS procedure

Totally, 41 consecutive patients (mean age 64 ± 11 years, 34 males) with an ICD (single‐chamber = 22, dual‐chamber = 15, resynchronization therapy = 4) enrolled between October 2013 and April 2015 were the subject to the current analysis. In the NICM group, the underlying etiology was as follows: 4 patients had HCM, 3 patients had LBBB‐induced CM and 3 remaining patients had DCM. During NIPS, VT/VF was induced in 8 (20%) patients with ICM. Seven (88%) patients had monomorphic VT (MMVT) and 1 (12%) patient had polimorphic VT induced. Five patients had their MMVT induced using 3 extrastimuli, in 2 patients two extrastimuli was enough to induce MMVT, and one polymorphic VT was induced with two extrastimuli. All NICM patients were noninducible at NIPS. All patients completed NIPS protocol and there were no major adverse events during the procedure. Demographic and clinical characteristics of patients who had VT/VF versus those who had no VT/VF induced at NIPS are compared in Table 1. There were no statistically significant differences between patients with or without VT/VF induction during NIPS.

**TABLE 1:** Comparison of NIPS‐inducible and NIPS‐noninducible patients.

	VT/VF at NIPS (*n* = 8)	No VT/VF at NIPS (*n* = 33)	*p*‐Value
Female	1 (13%)	6 (18%)	NS
Age (years)	62 ± 12	65 ± 11	NS
Age ≥65	3 (38%)	18 (55%)	NS
BMI	27 ± 3	28 ± 4	NS
LVEF (%)	28 ± 5	37 ± 15	NS
LVEDD (mm)	62 ± 5	57 ± 9	NS
NYHA class II	7 (88%)	22 (67%)	NS
NYHA class III	1 (13%)	4 (12%)	NS
QRS duration (ms)	123 ± 17	128 ± 26	NS
Ischemic etiology	8 (100%)	23 (70%)	NS
Nonischemic etiology	0	10 (30%)	NS
HCM	0	4 (12%)	NS
LBBB	2 (25%)	5 (15%)	NS
Prior MI	8 (100%)	22 (67%)	NS
PCI	5 (63%)	17 (52%)	NS
CABG	2 (25%)	5 (15%)	NS
AF	2 (25%)	12 (36%)	NS
GFR (mL/min)	75 ± 27	81 ± 24	NS
Syncope	1 (13%)	2 (6%)	NS
β‐blocker	7 (88%)	32 (97%)	NS
Amiodarone	2 (25%)	3 (9%)	NS
Sotalol	0 (0%)	1 (3%)	NS
ACE‐inhibitors	7 (88%)	24 (73%)	NS
Statins	7 (88%)	27 (82%)	NS

Abbreviations: ACE, angiotensin‐converting enzyme; AF, atrial fibrillation; BMI, body mass index; CABG, coronary artery bypass graft surgery; GFR, glomerular filtration rate; HCM, hypertrophic cardiomyopathy; LBBB, left bundle branch block; LVEF, left ventricular ejection fraction; LVEDD, left ventricular enddiastolic dimension; MI, myocardial infarction; NYHA, New York Heart Association; PCI, percutaneous coronary intervention.

### 3.2 Post‐NIPS ICD programming

In general, no changes in ICD programming were preformed after NIPS. Twenty‐seven (66%) patients had two detection zones programmed, whereas single zone was programmed in 7 (17%) and three zones were programmed in the remaining 7 (17%) patients. Mean VT zone was programmed at 173 ± 9 bpm and mean VF zone was programmed at 220 ± 18 bpm.

### 3.3 Endpoint occurrence during the 5‐year follow‐up

**FIGURE 1:**
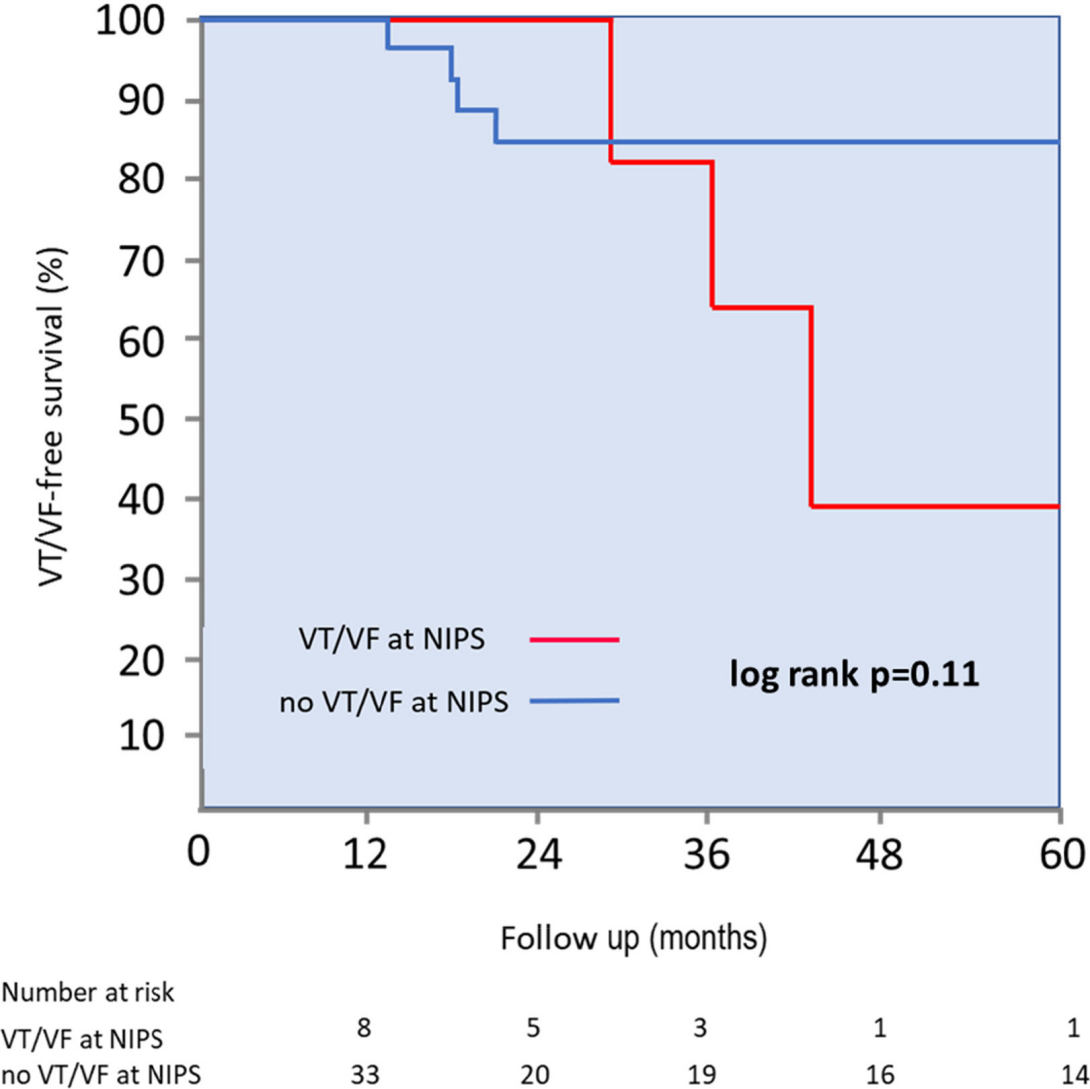
Kaplan–Meier VT/VF survival curves. NIPS, noninvasive programmed stimulation; VF, ventricular fibrillation; VT, ventricular tachycardia.

The median follow‐up was 60 months. During the 5‐year follow‐up, the VT/VF end‐point occurred in 7 (17%) patients. All of these episodes were MMVT. All these patients had underlying ischemic etiology. The difference between NIPS‐inducible versus NIPS‐noninducible patients regarding VT/VF occurrence did not meet statistical significance (38% vs. 12%, log rank test *p* = .11) (Figure 1). There was no statistically significant difference between cycle lengths of VT induced at NIPS and those that occurred spontaneously during observation (292 ± 27 vs. 285 ± 32 ms, *p* = .8). Also, there was no statistically significant difference between the induction of VT/VF with 3 versus 2 exstrastimuli during the NIPS and follow‐up VT recurrence (40% vs. 33%, *p* = .3).

**FIGURE 2:**
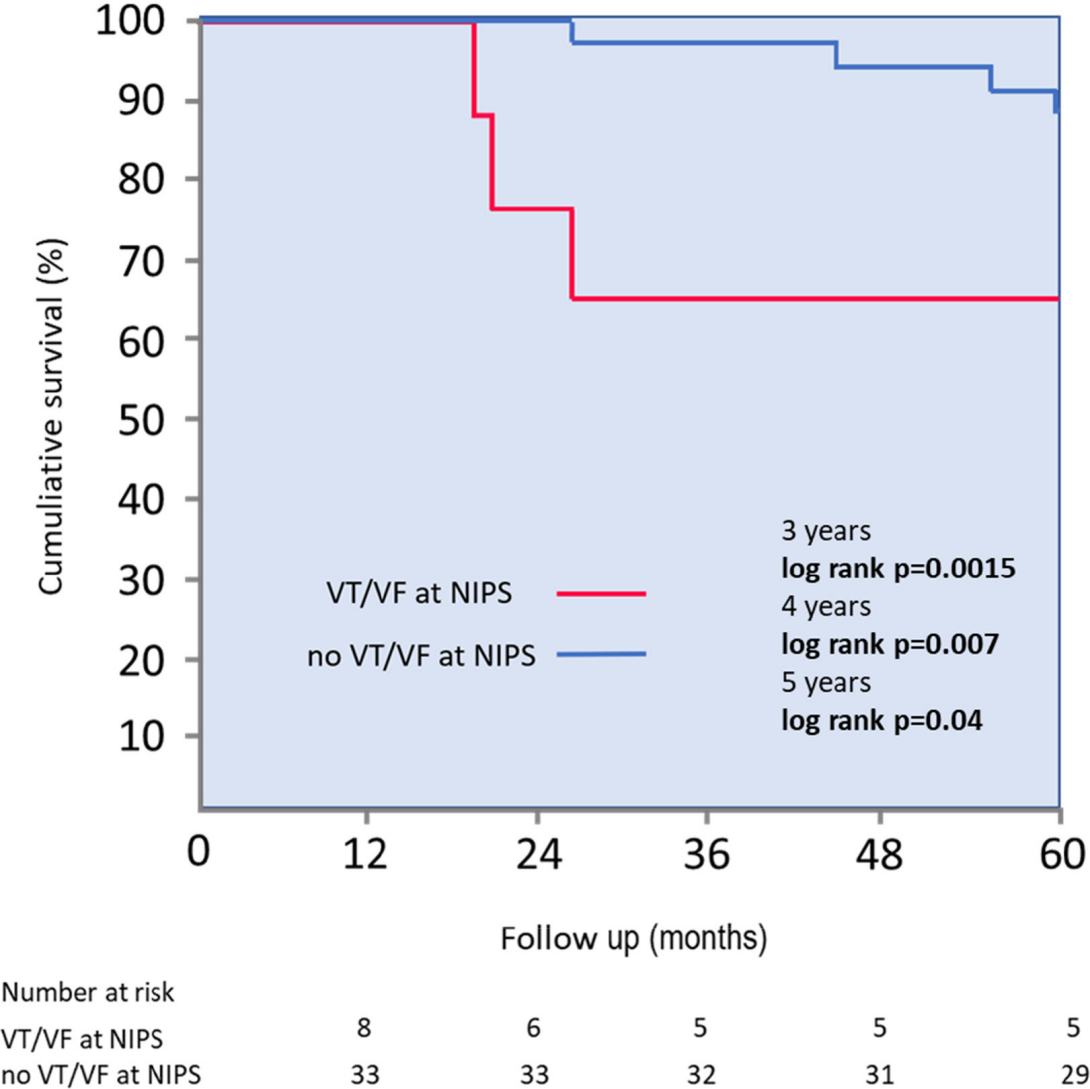
Kaplan–Meier cumulative survival curves. NIPS, noninvasive programmed stimulation; VF, ventricular fibrillation; VT, ventricular tachycardia.

**FIGURE 3:**
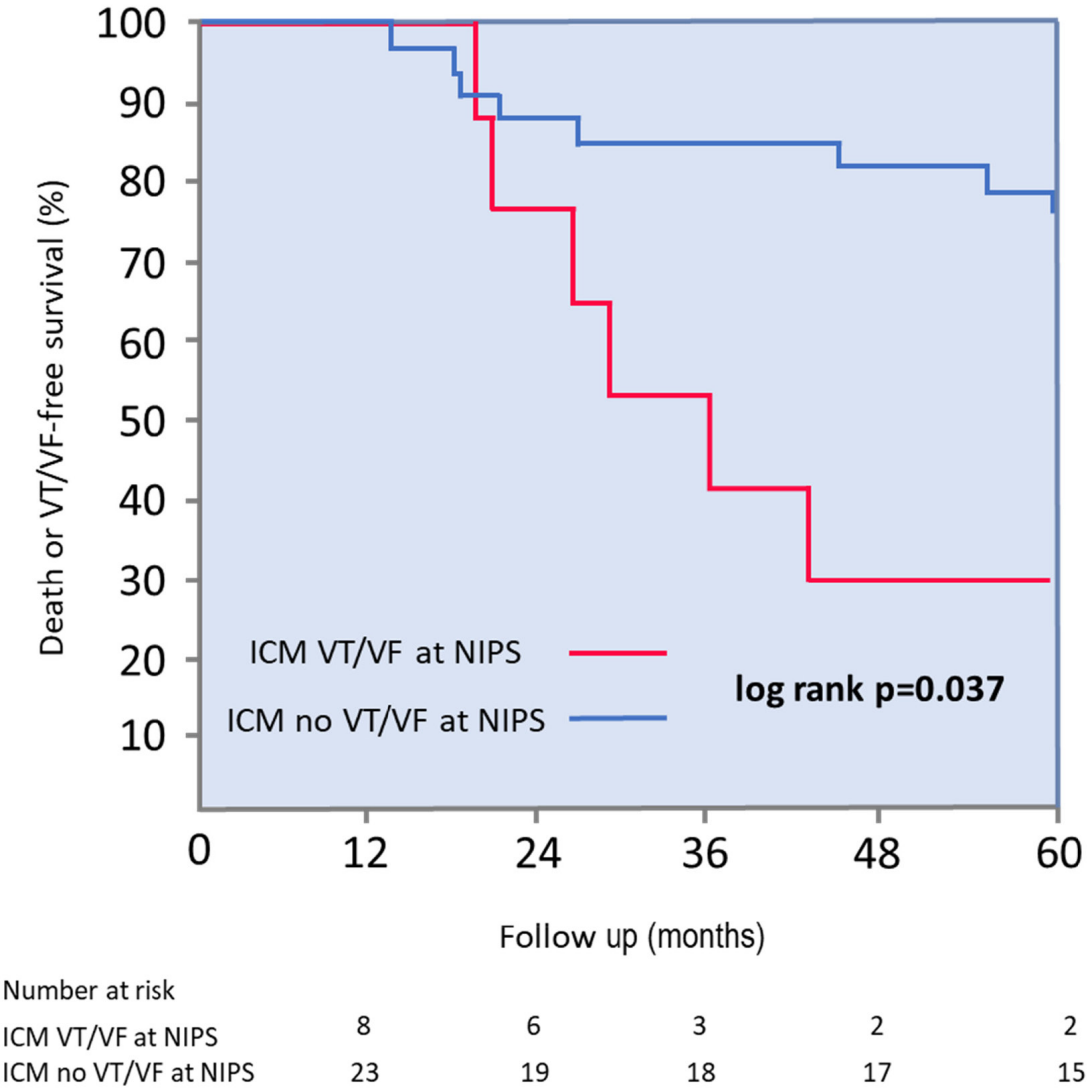
Kaplan–Meier death or VT/VF survival curves for ICM patients. ICM, ischemic cardiomyopathy; NIPS, noninvasive programmed stimulation; VF, ventricular fibrillation; VT, ventricular tachycardia.

Seven patients died during the follow‐up. After 5 years mortality rate was significantly higher in patients who were inducible at NIPS (38% vs. 12%, *p* = .043). The difference in mortality was most remarkable at 3 years (38% vs. 3%, *p* = .0015) and 4 years (38% vs. 6%, *p* = .007) (Figure 2). Inducibility of VT/VF at NIPS had sensitivity of 43%, specificity of 85%, positive predictive value of 38% and negative predictive value of 88% for occurrence of death during the follow‐up. In the ICM group an occurrence of secondary composite endpoint consisting of VT/VF event or death, which occurred in 14 patients, was also more frequent in NIPS‐inducible group (75% vs. 35%, log‐rank *p* = .037) (Figure 3).

Comparison of patients with versus without VT/VF during the follow‐up is presented in Table 2. There were no statistically significant differences between these groups of patients. Comparison of patients who died during the follow‐up versus survivors is presented in Table 3.

**TABLE 2:** Comparison of patients with VT/VF episode during the follow‐up and without VT/VF episode.

	VT/VF during FU *N* = 7	No VT/VF during FU *N* = 34	*p*‐Value
VT/VF at NIPS	3 (43%)	5 (15%)	NS
Female	1 (14%)	6 (18%)	NS
Age (years)	65 ± 13	64 ± 11	NS
Age ≥65	4 (57%)	17 (50%)	NS
BMI	27 ± 3	28 ± 4	NS
LVEF (%)	29 ± 5	36 ± 15	NS
LVEDD (mm)	63 ± 5	57 ± 9	NS
NYHA class II	5 (71%)	24 (73%)	NS
NYHA class III	2 (28%)	3 (9%)	NS
QRS duration (ms)	127 ± 18	127 ± 26	NS
Ischemic etiology	7 (100%)	24 (71%)	NS
Nonischemic etiology	0	10 (29%)	NS
HCM	0	4 (12%)	NS
LBBB	3 (43%)	4 (12%)	NS
Prior MI	7 (100%)	23 (68%)	NS
MI during the follow‐up	0	2 (6%)	NS
PCI	3 (43%)	19 (56%)	NS
CABG	2 (29%)	5 (15%)	NS
AF	2 (29%)	12 (35%)	NS
GFR (mL/min)	74 ± 18	81 ± 26	NS
Syncope	0	3 (9%)	NS
β‐blocker	7 (100%)	32 (94%)	NS
Amiodarone	2 (29%)	3 (9%)	NS
Sotalol	0	1 (3%)	NS
ACE inhibitors	4 (57%)	27 (79%)	NS
Statins	7 (100%)	27 (79%)	NS

Abbreviations: ACE, angiotensin‐converting enzyme; AF, atrial fibrillation; BMI, body mass index; CABG, coronary artery bypass graft surgery; GFR, glomerular filtration rate; HCM, hypertrophic cardiomyopathy; LBBB, left bundle branch block; LVEF, left ventricular ejection fraction; LVEDD, left ventricular enddiastolic dimension; MI, myocardial infarction; NYHA, New York Heart Association; PCI, percutaneous coronary intervention; VF, ventricular fibrillation; VT, ventricular tachycardia.

**TABLE 3:** Comparison of patients who died during the follow‐up versus survivors.

	Death during the follow‐up (*n* = 7)	Survivors (*n* = 34)	*p*‐Value
Female	2 (29%)	5 (15%)	NS
Age (years)	71 ± 6	63 ± 11	NS
Age ≥65	6 (86%)	15 (44%)	NS
BMI	28 ± 3	28 ± 4	NS
LVEF (%)	25 ± 5	37 ± 13	.043
LVEDD (mm)	62 ± 7	57 ± 9	NS
NYHA class II	6 (86%)	23 (68%)	NS
NYHA class III	1 (14%)	4 (12%)	NS
QRS duration (ms)	128 ± 29	127 ± 24	NS
Ischemic etiology	7 (100%)	24 (71%)	NS
Nonischemic etiology	0	10 (29%)	NS
HCM	0	4 (12%)	NS
LBBB	0	7 (21%)	NS
Prior MI	6 (86%)	24 (71%)	NS
MI during the follow‐up	0	2 (6%)	NS
PCI	6 (86%)	16 (47%)	NS
CABG	1 (14%)	6 (18%)	NS
AF	4 (57%)	10 (29%)	NS
GFR (ml/min)	65 ± 24	83 ± 24	NS
Syncope	0	3 (9%)	NS
β‐blocker	6 (86%)	33 (97%)	NS
Amiodarone	2 (29%)	3 (9%)	NS
Sotalol	0 (0%)	1 (3%)	NS
ACE‐inhibitors	6 (86%)	25 (74%)	NS
Statins	6 (86%)	28 (82%)	NS

Abbreviations: ACE, angiotensin‐converting enzyme; AF, atrial fibrillation; BMI, body mass index; CABG, coronary artery bypass graft surgery; GFR, glomerular filtration rate; HCM, hypertrophic cardiomyopathy; LBBB, left bundle branch block; LVEF, left ventricular ejection fraction; LVEDD, left ventricular enddiastolic dimension; MI, myocardial infarction; NYHA, New York Heart Association; PCI, percutaneous coronary intervention.

## 4 DISCUSSION

The main finding of the present study is that inducibility of VT/VF at NIPS is associated with increased mortality and occurrence of composite endpoint consisting of VT/VF occurrence and death during the long‐term follow‐up in patients who received an ICD for primary prevention indication.

### 4.1 Prognostic value of NIPS for the prediction of future VT/VF

Data on prognostic value of NIPS in primary prevention ICD recipients are scarce and only a few nonprospective studies on NIPS were published.^4^, ^5^, ^6^, ^7^ NIPS has been initially used for testing of detection and therapy features of ICDs.^4^, ^10^ In the recent years NIPS was implemented as a method allowing to identify patients at high risk after catheter ablation of VT.^11^, ^12^, ^13^, ^14^, ^15^ Induction rates of VT/VF at NIPS varied between these previous studies. In one study dealing with VT/VF survivors induction rates at NIPS were high (>85%), however patients enrolled in that study underwent multiple NIPS testing attempts.^4^ In contrast, another study performed in patients with NICM showed that despite more aggressive burst stimulation protocols at final stages of NIPS (burst pacing up to 250 ms), the induction rate at NIPS was low (14%).^6^ This stays in line with our findings—in our study none of NICM primary prevention‐ICD patients had VT/VF induced during NIPS using 12‐step protocol. This suggest that VT/VF inducibility by NIPS may highly depend on the etiology and patients with well‐defined substrate such as post‐myocardial scar may have higher inducibility rate (Table 4).

**TABLE 4:** Comparison of patients with nonischemic (NICM) and ischemic (ICM) etiology.

	NICM (*n* = 10)	ICM (*n* = 31)	*p*‐Value
Female	3 (30%)	4 (13%)	NS
Age (years)	58 ± 13	66 ± 10	NS
Age ≥65	3 (30%)	18 (58%)	NS
BMI	27 ± 5	28 ± 5	NS
LVEF (%)	45 ± 18	32 ± 13	.03
LVEDD (mm)	53 ± 9	59 ± 8	NS
NYHA class II	4 (40%)	25 (81%)	NS
NYHA class III	2 (20%)	3 (10%)	NS
QRS duration (ms)	132 ± 22	125 ± 27	NS
HCM	4 (40%)	0	.002
DCM	3 (30%)	0	NS
LBBB	3 (30%)	4 (13%)	NS
PCI	0	22 (71%)	.0001
CABG	0	7 (23%)	NS
AF	3 (30%)	11 (35%)	NS
GFR (mL/min)	92 ± 21	76 ± 24	NS
Syncope	1 (10%)	2 (6%)	NS
β‐blocker	10 (100%)	29 (94%)	NS
Amiodarone	0	5 (16%)	NS
Sotalol	0	1 (3%)	NS
ACE‐inhibitors	7 (70%)	24 (77%)	NS
Statins	6 (60%)	28 (90%)	NS

Abbreviations: ACE, angiotensin‐converting enzyme; AF, atrial fibrillation; BMI, body mass index; CABG, coronary artery bypass graft surgery; GFR, glomerular filtration rate; HCM, hypertrophic cardiomyopathy; LBBB, left bundle branch block; LVEF, left ventricular ejection fraction; LVEDD, left ventricular end‐diastolic dimension; NYHA, New York Heart Association; PCI, percutaneous coronary intervention.

### 4.2 NIPS result and mortality

The predictive value of NIPS has been shown in patients after catheter ablation of VT, and induction of clinical VT at NIPS had highest predictive value for VT/VF occurrence and also for mortality.^11^ Inducibility at the end of catheter ablation was associated with higher VT/VF occurrence and increased mortality in another study.^16^ The mechanism leading to higher mortality in patients who are inducible at NIPS is probably a composite of alterations in electrophysiological function, hemodynamic function and molecular changes interacting with the underlying substrate for VT/VF. Thus, induction of VT/VF during NIPS can probably identify some these increased‐ or high‐risk myocardial substrates. It is evident that more research is needed to help further understand how the presence of inducible VT/VF substrate, revealed using NIPS, may be contributing to mortality risk during the long‐term follow‐up.

### 4.3 Protocol of NIPS

In contrast to some other studies, our study used unified and strict NIPS protocol.^8^ Lack of standardized protocol was one of the limitations of earlier trials^4^, ^5^, ^6^, ^7^, ^8^, ^9^, ^10^, ^11^, ^12^, ^13^, ^14^, ^15^, ^17^ and significant divergence in methodology between past and recent studies—from ultra‐rapid 20 ms bursts to single, double, and triple extrastimuli delivered to refractoriness at drive trains of 600 and 400 ms. Thus, comparison of results of NIPS‐related studies may be difficult.^15^ In the presented study most patients required three extrastimuli for induction of MMVT and this can suggest the need of conducting a complete protocol in order to increase sensitivity of NIPS.

### 4.4 Clinical implications

In day‐to‐day practice, NIPS results are used for the evaluation of effectiveness of ablative treatment of VT.^12^, ^13^, ^14^, ^15^ Induction of MMVT at NIPS may be possibly used for identification of those patients who could benefit from prophylactic VT ablation well‐defined scar substrate for VT in NIPS‐inducible subjects can be the best target for preventive VT ablation. Future ablative treatment of VT/VF may also consist of more effective, safe and noninvasive techniques,^18^ allowing implementation of VT/VF ablation in more preventive manner. Thus, the usage of NIPS for selecting patients for prophylactic ablation can be possibly of value, however, this requires further studies in more homogenous groups of patients.

Other interventions can also impact mortality in patients with ICDs. For example, MADIT‐RIT study showed a mortality benefit and lower incidence of inappropriate ICD therapies achieved by ICD programming of zones >200 bpm. This approach however can be limited when patients are vulnerable to VTs below such high detection zones and cycle lengths of VT induced at NIPS has been used for programming purposes in one study.^12^, ^13^ Weather patients equipped with ICD who had VT/VF induced at NIPS can benefit from more intense HF treatment may require further determination.

The fact that all spontaneous VT/VF episodes occurred only in patients with ICM and in none of the patients with NICM may depict the already described fact that NICM patients with severe left ventricular dysfunction has less “pure” arrhythmic risk compared to ICM.^19^

### 4.5 Limitations

This study has some limitations. First of all, as the initial sample size of the NIPS‐ICD study was calculated for the total cohort of patients with primary and secondary ICD indication, the number of patients in the current analysis is small. Such a small sample size possibly has an impact on statistical comparisons in the presented study. Second, patients who were enrolled presented mixture of etiology (ischemic and nonischemic). Thirdly, the results of our study were mainly driven by the ICM sub‐cohort so it remains unknown if these results can be extrapolated to larger groups of NICM patients. Lastly, the number of primary endpoints during the follow‐up was low.

## 5 CONCLUSIONS

In primary prevention ICD recipients VT/VF induction at NIPS is associated with increased mortality and incidence of composite endpoint during the long‐term follow‐up. Noninducibility at NIPS may identify those who have lower probability of death during observation. Whether NIPS results may serve as an indicator for performing prophylactic therapeutic interventions such as ICD programming, catheter ablation, antiarrhythmic drug therapy or more aggressive HF therapy in particular primary prevention ICD sub‐cohorts needs to be examined in further studies.

## CONFLICT OF INTEREST STATEMENT

Authors declare no conflict of interests for this article.

## ETHICS STATEMENT

Study protocol was approved by the Ethics Committee of the Regional Medical Chamber in Rzeszów.

## CLINICAL TRIAL REGISTRATION

The study was registered (ClinicalTrials.gov ID: NCT02373306) and a detailed study protocol was published.

## PATIENT CONSENT STATEMENT

Written consent was obtained from all patients.

## Data Availability

The data that support the findings of this study are available on request from the corresponding author, Piotr Futyma.
